# Phytochemical Supplementation in Grazing Dairy Cows: Effects on Milk Production, Metabolic Status, Hematology, and Postpartum Ovarian Activity

**DOI:** 10.1155/vmi/2399561

**Published:** 2026-08-21

**Authors:** Manuela Comesaña, Ana Meikle, Diego Mattiauda, Marisela Arturo-Schaan, Aline C. Dall-Orsoletta, Rodrigo Vivián, Gretel Ruprechter, María de Lourdes Adrien

**Affiliations:** ^1^ Department of Veterinary and Agricultural Sciences, Faculty of Veterinary Medicine, Universidad de la República, Route 3 km 363, Paysandú 60000, Uruguay, universidad.edu.uy; ^2^ Department of Clinics and Veterinary Hospital, Faculty of Veterinary Medicine, Universidad de la República, Route 8 km 18, Montevideo, Uruguay, universidad.edu.uy; ^3^ Department of Animal Production and Pastures, Faculty of Agronomy, Universidad de la República, Av. Garzón 780, Montevideo 12900, Uruguay, universidad.edu.uy; ^4^ CCPA France, CCPA Group, ZA du Bois de Teillay, Janzé 35150, France

**Keywords:** animal health, dairy cows, transition period

## Abstract

This study evaluated the effects of three treatments in dairy cows under pasture‐based systems during the transition and early lactation periods: one group supplemented with a phytochemical mix (polyphenols, curcuminoids, piperine, and trans‐cinnamaldehyde, PHY), another group supplemented with monensin (MON), and a third group with no additive, used as the control (CTL). Sixty Holstein cows (primiparous and multiparous) were assigned to these treatments from 30 days prepartum to 60 days postpartum. Productive, metabolic, endocrine, hematological, and reproductive parameters were analyzed. Results showed that both PHY and MON increased milk yield in primiparous cows compared to CTL, whereas no treatment differences were observed in multiparous cows. MON supplementation was associated with higher serum cholesterol concentrations, particularly in primiparous cows. Total leukocyte counts did not differ between treatments; however, a treatment effect was observed for monocytes in multiparous cows. Energy balance indicators (nonesterified fatty acids, beta‐hydroxybutyrate, and glucose) and calcium and phosphorus were not significantly influenced by treatments and remained within physiological ranges. In conclusion, both MON and the phytochemical mix supported adaptation during the transition period, although their effects differed by parity and physiological response. MON was more closely associated with lipid metabolism, while the phytochemical mix may have contributed to subtle immune‐related adjustments, warranting further investigation.

## 1. Introduction

Dairy production faces a growing demand to increase both milk and animal health, particularly during the transition period. This stage, which spans from 3 weeks before to 3 weeks after calving, is characterized by substantial metabolic and physiological adaptations. However, high‐producing dairy cows frequently experience a negative energy balance [1], which can predispose them to metabolic disorders and reduced reproductive efficiency [2, 3]. Therefore, strategies that support metabolic stability and immune competence during the transition period are critical to sustain herd productivity in pasture‐based systems [4].

The increased energy demands for milk production during early lactation are often not met by voluntary feed intake, leading to lipomobilization and altered hepatic metabolism [1]. These metabolic adjustments are associated with variations in blood metabolites, endocrine responses, and immune function, which together influence the cow’s ability to adapt to the transition period. Improving metabolic resilience during this stage is therefore likely to enhance disease resistance and reproductive performance [5].

Among the nutritional strategies used to support metabolic adaptation, monensin (MON) has been widely adopted due to its capacity to modify ruminal fermentation toward increased propionate production, thereby enhancing gluconeogenic potential and potentially reducing the severity of negative energy balance in early lactation cows [6, 7]. MON supplementation has been associated with the reduction in incidence of metabolic disorders such as ketosis [8], and the improvement of body condition and milk production [9, 10]. However, concern regarding the use of ionophores in dairy production has increased, prompting interest in natural feed additives, such as phytochemicals (plant‐derived compounds) that may offer similar physiological benefits without antimicrobial properties, and thereby improving the overall resilience of ruminants under challenging environmental conditions.

Phytochemicals are bioactive compounds produced by plants, including polyphenols, curcuminoids, piperine, and cinnamaldehyde, which have been associated with antioxidant, anti‐inflammatory, and rumen‐modulatory properties [11]. Recent studies have suggested that phytochemical mixes may improve metabolic stability, immune balance, and overall adaptation to early lactation [12, 13]. However, most research has been conducted in confined systems, and evidence under mixed pasture supplementation is limited.

Most previous research has been conducted in confined systems with total mixed rations (TMR) [14–16], which differs from dairy mixed systems, where up to 50% of the diet comes from grazing [17]. Moreover, many studies have focused on mid or late‐lactation cows, without evaluating early lactation which is the critical stage in which the transition to a new diet entails a higher risk of subclinical acidosis and when feed additives are commonly used. Therefore, the objective of this study was to compare a phytochemical mix (PHY) with MON and a control diet (CTL) in Holstein dairy cows grazing a mixed system during the transition and early lactation periods. We hypothesized that PHY would produce comparable productive and metabolic responses to MON, while also potentially affecting hematological patterns associated with immune function, without compromising reproductive performance.

## 2. Materials and Methods

The use and procedures with animals were approved by the Ethics Committee of the University of the Republic, Montevideo, Uruguay (CHEA Protocol No. 1330, file number: Exp. 020300‐000055‐22).

### 2.1. Animals and Experimental Design

The experiment was carried out at the Estación Experimental “Dr. Mario A. Cassinoni,” Facultad de Agronomía, Universidad de la República, Paysandú, Uruguay, from February to June 2022. A total of 60 Holstein cows (24 primiparous and 36 multiparous cows) were enrolled and distributed in three treatments. Cows were blocked by parity, estimated calving date, and body weight and then randomly assigned to treatments from −30 to 60 postpartum days. The treatments were as follows: (1) PHY—50 g/cow/day of a phytochemical mix (polyphenols ≤ 450 ppm, curcuminoids ≤ 280 ppm, piperine ≤ 15 ppm, and trans‐cinnamaldehyde ≤ 1000 ppm; formulation and dosage provided and recommended by CCPA Group, France), (2) MON—0.3 g/cow/day of sodium MON, and (3) CTL—control, with no additive. Additives were incorporated and mixed into the TMR, in prepartum TMR (anionic diet) and postpartum TMR. At the beginning of the study, body condition score (BCS) averaged 3.36 ± 0.27 in primiparous and 3.24 ± 0.31 in multiparous cows (mean ± SD), and live weight (LW) averaged 581 ± 14 and 655 ± 12 kg, respectively.

From the total of 60 cows enrolled at the beginning of the study, 50 healthy cows completed the study. Ten cows were removed due to anaplasmosis (1), leukosis (1), teat disease and teat‐cistern obstruction affecting milk production (3), metritis and retained placenta (3), and severe mastitis affecting general condition (2). Therefore, the results reported correspond to 18 PHY cows (5 primiparous and 13 multiparous), 16 MON cows (6 primiparous and 10 multiparous), and 16 CTL cows (7 primiparous and 9 multiparous).

#### 2.1.1. Animal Handling and Feeding

Cows in the prepartum period were confined in a compost bedding barn with natural ventilation, fans, and sprinklers. Cows were placed in pens separated by parity (4 cows/pen), with ad libitum water, and received a TMR diet containing a mineral premix plus the assigned treatment (Table 1). The TMR diet was offered collectively to each pen.

**TABLE 1:** Ingredients and chemical composition of the total mixed ration and partially mixed ration in prepartum and postpartum periods.

Ingredients (% DM)	TMR prepartum	TMR postpartum
Corn silage	38.8	30.6
Barley straw	31.4	—
Alfalfa hay	—	13.6
Soybean meal	16.2	13.6
Corn grain	4.37	26.4
Wheat bran	4.42	—
Barley grain	—	7.65
Calcium carbonate	1.36	1.69
Sodium chloride	—	0.68
Baking soda	—	0.66
Magnesium oxide	—	0.66
Monocalcium phosphate	—	0.66
Sulfur flowers	—	1.74
Semitin wheat	—	1.74
^1^Mineral–vitamin supplement	3.45	0.32
*Chemical composition*		
Organic matter (%DM)	90.6 ± 1.34	91.3 ± 0.44
Crude protein (%DM)	10.8 ± 1.48	12.8 ± 0.96
Neutral detergent fiber (%DM)	44.2 ± 2.51	26.8 ± 3.44
Acid detergent fiber (%DM)	25.9 ± 1.02	14.0 ± 1.50
Digestible energy (Mcal/kg DM)	2.86 ± 0.01	3.07 ± 0.01
Net lactation energy (Mcal/kg DM)	1.47 ± 0.01	1.67 ± 0.01

^1^Vitamin–mineral supplement in the prepartum: −9831 meq/kg, 6% Mg, 0.20% K, 2% Na, 16% Cl, 10% S, 10% Ca, 250 mg/kg Cu, 8.0 mg/kg Se, 1200 mg/kg Zn, 9 mg/kg I, 2.4 mg/kg Co, 210,000 IU/kg, vitamin A, 90,000 IU/kg, vitamin D, 2400 IU/kg, vitamin E. Postpartum: 20,000 mg/kg Zn, 4000 mg/kg Cu, 120 mg/kg Se, 160 mg/kg I, 40 mg/kg Co, 1,200,000 IU/kg vitamin A, 240,000 IU/kg vitamin D3, 8400 IU/kg vitamin E. DTM_EN L_: 1909 − (0.017 × FA).

An anionic diet was supplied prepartum (DCAD = −140 mEq/kg DM) and monitored weekly via urine pH. Prepartum dry matter intake (DMI) was recorded daily. Prepartum DMI in primiparous cows was 1.88%, 1.98%, and 2.06% LW for MON, PHY, and CTL, respectively, while multiparous cows consumed 2.17%, 2.14%, and 2.21% LW for MON, PHY, and CTL, respectively. According to the final consumption of TMR, the dose of additives consumed by the animals was 46.4 g/cow/day of phytochemical mix and 0.27 g/cow/day of MON.

In the postpartum period, cows grazed pasture and received partial supplementation with the TMR shown in Table 1. Each treatment grazed in separate plots, and forage allocation was adjusted weekly using the double sampling technique [18]. Postpartum DMI was 3.68% LW in primiparous cows and 3.83% LW in multiparous cows. Pasture availability ranged from 1764 ± 100 to 2936 ± 826 kg DM, with pasture height ranging from 15.0 ± 1.28 to 28.8 ± 4.50 cm. The average pasture offered was 28.1 ± 4.71 kg DM/cow/day.

The methodology for determining the chemical composition of the TMR diet mentioned previously in Table 1 is described in the work of Gonzalez‐Chappe et al., [19].

#### 2.1.2. Determinations and Sampling

All determinations were made from 30 days before calving to 60 days in milk (DIM).

Cows were milked twice by day (at 4:00 a.m. and 4:00 p.m.), and individual production was recorded daily using automatic milk meters synchronized with the software of DairyPlan® (Gea Farm Technologies, Düsseldorf, Germany).

BCS was evaluated weekly, and the assessment was made by a single observer using a scale from 1 to 5 [20], and the LW was registered monthly.

Two reproductive evaluations were performed at 23 ± 1 and 44 ± 3 days postpartum. These consisted of assessing uterine involution and identifying the presence of the corpus luteum (CL) using ultrasound. The ultrasound device used was an Aloka SSD 500 with a 5 MHz linear probe.

Blood samples were obtained from the coccygeal vein twice weekly after the morning milking, prior to feeding, using one needle per animal, in vacuum tubes with EDTA (blood count), sodium fluoride (glucose), and without anticoagulant (metabolites and hormones) (VACUETTE® tubes). To obtain serum and plasma, the tubes were centrifuged at 3500 rpm for 10 min and stored at −20°C until the determination of metabolites (NEFA, BHB, cholesterol, calcium, phosphorus, and glucose) and hormones (progesterone and insulin). Serum metabolites and hormones were determined in the Laboratorio de Endocrinología y Metabolismo Animal of the Facultad de Veterinaria, Universidad de la República, Uruguay. The metabolites were determined, weekly, by spectrophotometry using an automatic analyzer BA200 (©Biosystems SA Barcelona, Spain) with commercial kits. Also, commercial control kits were used. For NEFA, the coefficient of variation (CV) for the single assay (1.92 mmol/L) was 5%; for BHB, control 1 (0.3 mmol/L) was 7% and control 2 (1.65 mmol/L) was 5%; for cholesterol, control 1 (4.3 mmol/L) was 3% and in control 2 (6.96 mmol/L) was 2%; and for glucose, control 1 (5.01 mmol/L) was 2% and control 2 (14.7 mmol/L) was 3%, concluding that all were less than 10%. Serum progesterone (P4) was analyzed by solid‐phase radioimmunoassay (RIA), using a commercial kit (MP Biomedicals, Los Angeles, CA, USA) as reported by Ruprechter et al. [21]. The sensitivity of the assay was 0.11 ng/mL, with an interassay CV coefficient for low control (0.7 ng/mL) of 18.5% and for medium control (4.8 ng/mL) of 17%. The resumption of ovarian activity was defined as the day when the concentration of progesterone was greater than 1 ng/mL in two consecutive blood serum samples. For this analysis, only cows that presented CL were analyzed.

Serum insulin concentrations were determined using the INS‐IRMA kit (DIAsource ImmuneAssays S.A., Belgium); the interassay CV was 5% for control 1 (19.9 μIU/mL) and 6% for control 2 (56.2 μIU/mL).

The determination of the white blood cells (WBC), red blood cells (RBC), hematocrit (HCT), mean corpuscular volume (MCV), mean corpuscular hemoglobin (MCH), hemoglobin (HGB), mean corpuscular hemoglobin concentration (MCHC), red cell distribution width (RDW), and platelets (PLT) was carried out on the days of blood extraction in a clinical laboratory in Paysandú, Uruguay (Laboratorio Alfa), using a counter 19 hematological counter (Wiener lab) whose measurement principle was by electrical impedance plus colorimetry, with subsequent validation by blood smear [22].

#### 2.1.3. Statistical Analysis

All data were analyzed using SAS Studio (Version 9.04.0, SAS Institute Inc., Cary, NC, USA). Normality was assessed using PROC UNIVARIATE. The gamma distribution was used for BCS analysis because it did not exhibit a normal distribution. Milk production data were analyzed by parity using the GLIMMIX procedure, including treatment effects as fixed effects, cow as a random effect, and body weight, DIM, and DIM2 as covariates. BCS, metabolites, and complete blood count data were analyzed by parity from 30 days prepartum to 60 DIM using the GLIMMIX procedure, including treatment, period, and their interaction effects as fixed effects in the statistical model. BCS at the start of the experiment was used as a covariate. A covariance structure of type: ar(1) was used, considering cow (treatment). For the analysis, time was considered in periods of days. Post hoc analyses were performed using the Tukey–Kramer test. Differences were considered significant when *p* < 0.05 and a trend when *p* ≤ 0.10. Insulin was analyzed as in the previous studies, without considering baseline body condition as a covariate. The percentage of cows that exhibited a CL was analyzed using binomial probability with the GLIMMIX procedure, considering treatment, days postpartum, and their interaction as fixed effects. The day of resumption of ovarian cyclicity was analyzed using the GLIMMIX procedure, with treatment as a fixed effect.

## 3. Results

### 3.1. Milk Production

The use of additives (MON and PHY) in primiparous cows resulted in higher milk yield than CTL cows (*p* = 0.01, Table 2).

**TABLE 2:** Milk yield in primiparous and multiparous cows treated with monensin (MON), mix of phytochemicals (PHY) or control (CTL).

	Treatment					*p* value		
	CTL	MON	PHY	SEM	T	LW	DIM	DIM^2^
Primiparous (kg/d)	26.5^b^	28.5^a^	28.7^a^	0.52	0.01	0.01	< 0.0001	< 0.0001
Multiparous (kg/d)	36.9^xy^	37.7^x^	36.0^y^	0.48	0.07	< 0.0001	< 0.0001	< 0.0001

*Note:* Different letters in rows indicate significant differences between treatment. a vs b, *p* < 0.05; x vs y, *p* < 0.1. Fixed effects: treatment (T). Live weight (LW), days in milk (DIM), and days in squared milk (DIM^2^) were covariate.

In multiparous cows, milk yield tended to differ among treatments (*p* < 0.01, Table 2), with MON cows tending to produce more milk than PHY cows.

### 3.2. Body Condition Score and Blood Metabolites

#### 3.2.1. Primiparous

The results of BCS and blood metabolites in primiparous cows are presented in Table 3.

**TABLE 3:** Body condition score (BCS) and metabolite concentrations throughout the period in control (CTL, *n* = 7), monensin (MON, *n* = 6), and mix of phytochemicals (PHY, *n* = 5) primiparous cows.

Parameter	Treatment				*p* value			
	CTL	MON	PHY	SEM	T	Per	T ∗ Per	BCSi
BCS	3.08^b^	3.21^a^	3.10^ab^	0.04	0.05	< 0.0001	0.30	0.04
NEFA (mM)	0.36	0.39	0.39	0.04	0.82	0.006	0.51	0.68
BHB (mM)	0.62	0.65	0.52	0.04	0.11	< 0.0001	0.67	0.01
Cholesterol (mM)	2.59^b^	3.09^a^	2.60^b^	0.13	0.03	< 0.0001	0.68	0.31
Glucose (mM)	3.53	3.66	3.62	0.06	0.30	< 0.0001	0.94	0.23
Calcium (mM)	2.01	2.04	2.05	0.02	0.65	0.03	0.10	0.97
Phosphorus (mM)	2.36	2.39	2.38	0.07	0.94	0.56	0.63	0.54
Ca/P	0.88	0.87	0.88	0.03	0.99	0.32	0.66	0.41

*Note*: Different letters in rows indicate significant differences between treatments. a vs b, *p* < 0.05; x vs. y is when p is less than 0.1 but greater than 0.05. BHB: beta‐hydroxybutyrate; Ca/P: calcium/phosphorus. Fixed effects: treatment (T), period (Per), and their interaction. BCS at the initiation of the treatments is the covariable (BCSi).

Abbreviation: NEFA, nonesterified fatty acids.

**FIGURE 1:**
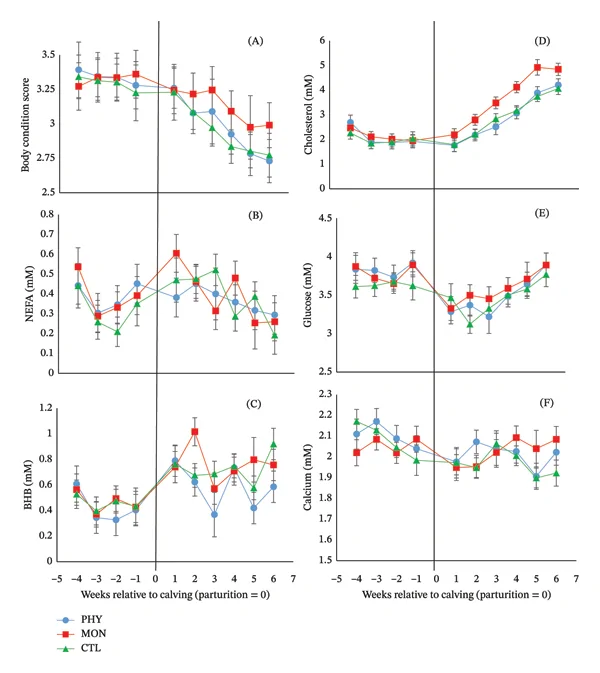
Evolution of body condition score (BCS, (A)), nonesterified fatty acids (NEFA, (B)), beta‐hydroxybutyrate (BHB, (C)), cholesterol (D), glucose (E), and calcium (F) in mix of phytochemical (PHY), monensin (MON), and control (CTL) primiparous cows.

Body condition was affected by the treatment (*p* < 0.1), with higher values in MON cows compared to CTL, while PHY cows did not differ from the other treatments (Table 3). BCS decreased after calving in all groups (Figure 1A).

NEFA and BHB concentrations were not affected by the treatment; concentrations increased towards calving (Figure 1B,C, Table 3).

Cholesterol concentration was affected by treatment (*p* < 0.1), with greater values in MON cows compared with CTL and PHY (Table 3). Cholesterol decreased toward calving and increased after the first postpartum week (Figure 1D).

Glucose concentration was not affected by treatment (Table 3). Values decreased toward calving and increased after the third postpartum week in all groups (Figure 1E).

The concentration of calcium, phosphorus, and the relation Ca/P was not affected by the treatment (Table 3), but the period affected the concentration of calcium, having lower concentrations around the first week after calving (Figure 1F).

#### 3.2.2. Multiparous

The results of BCS and blood metabolites in multiparous cows are presented in Table 4.

**TABLE 4:** Body condition score (BCS) and metabolite concentrations throughout the period in control (CTL, *n* = 9), monensin (MON, *n* = 10), and mix of phytochemicals (PHY, *n* = 13) multiparous cows.

Parameter	Treatment				*p* value			
	CTL	MON	PHY	SEM	T	Per	T ∗ Per	BCSi
BCS (1‐5)	3.01	2.98	3.00	0.07	0.97	< 0.0001	0.58	0.26
NEFA (mM)	0.32	0.30	0.34	0.02	0.29	< 0.0001	0.47	0.01
BHB (mM)	0.52	0.54	0.53	0.03	0.67	< 0.0001	0.11	0.001
Cholesterol (mM)	2.73	2.90	2.54	0.13	0.14	< 0.0001	0.05	< 0.0001
Glucose (mM)	3.42	3.40	3.41	0.04	0.99	< 0.0001	0.53	0.31
Calcium (mM)	2.01	1.98	2.02	0.02	0.30	0.04	0.79	0.001
Phosphorus (mM)	2.16	2.13	2.12	0.06	0.85	0.04	0.35	0.62
Ca/P	0.97	0.96	0.98	0.03	0.89	0.13	0.50	0.64

*Note:* BHB: beta‐hydroxybutyrate, Ca/P: calcium/phosphorus. Fixed effects: treatment (T), period (Per), and their interaction. BCS at the initiation of the treatments is the covariable (BCSi).

Abbreviation: NEFA, nonesterified fatty acids.

Neither body condition nor metabolites were affected by the treatment, or the interaction between treatment and period.

**FIGURE 2:**
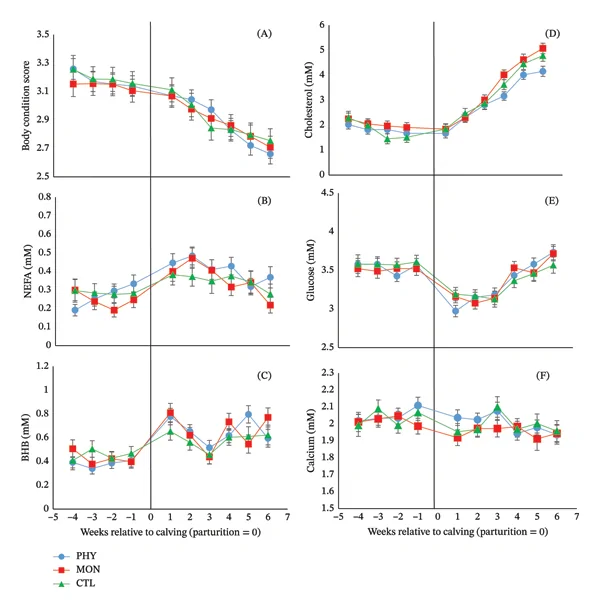
Evolution of body condition score (BCS, (A)), nonesterified fatty acids (NEFA, (B)), beta‐hydroxybutyrate (BHB, (C)), cholesterol (D), glucose (E), and calcium (F) in mix of phytochemical (PHY), monensin (MON), and control (CTL) multiparous cows.

BCS decreased toward calving in all groups. NEFA and BHB increased postpartum, with NEFA declining after the second postpartum week, and BHB decreasing after the first week, increasing slightly in the third week, and stabilizing thereafter (Figure 2B,C). Cholesterol and glucose increased after calving in all groups (Figure 2D and E). Calcium in the PHY and CTL groups increased during the third postpartum week and decreased thereafter, while in the MON group, it remained stable (Figure 2F).

### 3.3. Insulin

**FIGURE 3:**
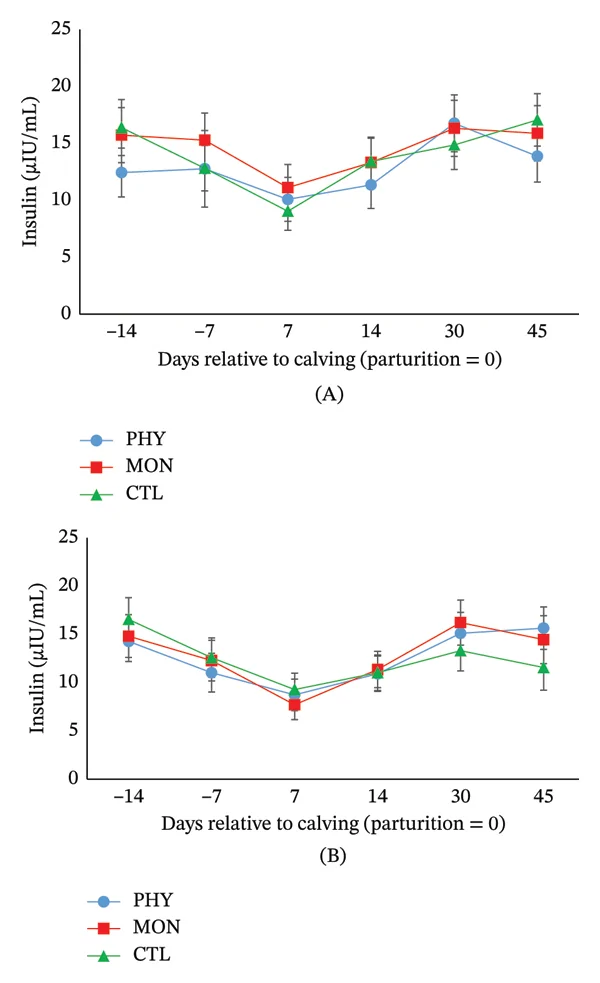
Evolution of insulin concentration in primiparous (A) and multiparous (B) cows in the mix of phytochemical (PHY), monensin (MON), and control (CTL) groups.

Insulin concentration was not affected by treatment (primiparous: CTL: 13.7, MON: 14.5, PHY 12.7 ± 1.1 uUI/mL and multiparous CTL: 12.2 MON: 12.5, PHY 12.4 ± 1.3 uUI/mL; mean ± SEM), but there was an effect on the period in primiparous and multiparous cows. Concentrations decreased towards calving and increased again after the second week of calving (Figure 3).

### 3.4. Hemogram Parameters

#### 3.4.1. Primiparous

The WBC and the individual parameters of the white line were not affected by the treatment (Table 5). There was an interaction between treatment and period in monocyte counts, explained by an increase in MON and in PHY cows at 50 days postpartum, which did not occur in control cows.

**TABLE 5:** White blood cell (WBC) total and differential counts, red blood cells (RBC), hemoglobin (HGB), hematocrit (HCT), mean corpuscular volume (MCV), mean corpuscular hemoglobin (MCH), mean corpuscular hemoglobin concentration (MCHC), red cell distribution width (RDW), platelets (PLT) in control (CTL, *n* = 7), monensin (MON, *n* = 6), and mix of phytochemicals (PHY, *n* = 5) primiparous cows.

Parameter		Treatment				*p* value		
	Reference values^∗^	CTL	MON	PHY	SEM	T	Per	T ∗ Per
WBC/μL	4900–12,000	12,511	12,332	12,572	1399	0.99	0.16	0.26
Lymphocytes/μL	1600–5600	5007	4385	4711	645	0.78	0.2	0.29
Neutrophils/μL	1800–6300	5936	6347	6313	746	0.9	0.15	0.49
Monocytes/μL	0–800	688	498	647	87	0.21	0.59	0.01
Eosinophils/μL	0–900	694	731	791	111	0.81	0.02	0.43
RBC (× 10^3^/μL)	5100–7600	5811	5783	5654	100	0.53	0.004	0.57
Hemoglobin (g/dL)	8.5–12.2	9.61	10.1	9.65	0.2	0.11	0.002	0.41
Hematocrit (%)	22–33	27.7^b^	29.8^a^	27.9^ab^	0.52	0.03	0.003	0.6
MCV (fL)	38–50	47.9^b^	51.8^a^	49.4^ab^	0.91	0.02	< 0.0001	0.9
MCH (pg)	14–18	16.5^b^	17.5^a^	17.0^ab^	0.15	0.001	0.001	0.43
MCHC (g/dL)	36–39	34.7	34.1	34.6	0.26	0.19	0.02	0.69
RDW (%)	15.5–19.7	18.2	18.5	18.4	0.16	0.32	0.03	0.55
PLT (× 10^3^/mL^3^)	100–800	352	345	329	24	0.8	0.002	0.64

*Note:* Different letters in rows indicate significant differences between treatment. a vs b, *p* < 0.05; x vs. y is when *p* is less than 0.1 but greater than 0.05. Fixed effects: treatment (T), period (Per), and the interaction.

^∗^[23].

The HCT, MCV, and MCH were affected by treatment, as MON had greater values than control cows (*p* < 0.1), while PHY cows did not differ from the rest (Table 5). All increased as lactation advanced.

#### 3.4.2. Multiparous

The WBC was not affected by treatment (Table 6). The treatment affected the count of monocytes (*p* = 0.01), as MON had greater counts than CTL cows (*p* < 0.1), while PHY cows did not differ from the other treatments (Table 6).

**TABLE 6:** White blood cell (WBC) total and differential counts, red blood cells (RBC), hemoglobin (HGB), hematocrit (HCT), mean corpuscular volume (MCV), mean corpuscular hemoglobin (MCH), mean corpuscular hemoglobin concentration (MCHC), red cell distribution width (RDW), platelets (PLT) in control (CTL, *n* = 9), monensin (MON, *n* = 10), and mix of phytochemicals (PHY, *n* = 13) multiparous cows.

Parameter		Treatment				*p* value		
	Reference values^∗^	CTL	MON	PHY	SEM	T	Per	T ∗ Per
WBC/μL	4900–12,000	11,482	15,462	12,827	1568	0.23	0.02	0.60
Lymphocytes/μL	1600–5600	4174	6573	5123	865	0.19	0.31	0.35
Neutrophils/μL	1800–6300	5690	6791	6264	509	0.36	0.06	0.90
Monocytes/μL	0–800	560^b^	991^a^	741^ab^	83	0.01	0.3	0.04
Eosinophils/μL	0–900	613	820	680	89	0.29	0.01	0.51
RBC (× 10^3^/μL)	5100–7600	5310	5277	5428	101	0.51	0.02	0.60
Hemoglobin (g/dL)	8.5–12.2	9.62	9.53	9.72	0.2	0.7	0.02	0.40
Hematocrit (%)	22–33	28.4	27.8	28.5	0.47	0.55	0.02	0.50
MCV (fL)	38–50	53.8	52.7	53.2	0.78	0.62	< 0.0001	0.75
MCH (pg)	14–18	18.2	18	18.1	0.2	0.92	0.25	0.62
MCHC (g/dL)	36–39	33.9	34.2	34.1	0.13	0.25	< 0.0001	0.44
RDW (%)	15.5–19.7	19.0	19.6	19.3	0.17	0.08	0.04	0.87
PLT (× 10^3^/mL^3^)	100–800	328	295	310	13	0.28	< 0.0001	0.83

*Note*: Different letters in rows indicate significant differences between treatment. a vs b, *p* < 0.05; x vs. y is when *p* is less than 0.1 but greater than 0.05. Fixed effects: treatment (T), period (Per), and the interaction.

^∗^[23].

None of the red blood parameters were affected by treatment (Table 6). There was an effect of the period on the RBC, HGB, HCT, MCV, MCHC, RDW, and PLTs, and all of them increased towards calving (Table 6).

### 3.5. Resumption of Ovarian Activity

#### 3.5.1. Days to First Ovulation According to P4 Levels

**FIGURE 4:**
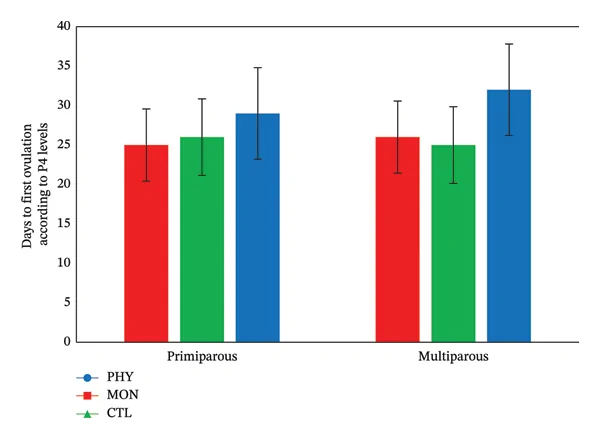
Days to first ovulation according to P4 levels in primiparous and multiparous cows in the mix of phytochemical (PHY), monensin (MON), and control (CTL) groups.

No significant differences were found between treatments in relation to the days of the first ovulation. Figure 4 shows the average of days to first ovulation in cows from the MON, PHY, and CTL groups. For primiparous cows, the average time to first ovulation was 25 ± 5 days in the MON group, 29 ± 8 days in the PHY group, and 26 ± 5 days in the CTL group. In multiparous cows, the averages were 26 ± 4 days for MON, 33 ± 4 days for PHY, and 25 ± 4 days for CTL.

#### 3.5.2. Percentage of Cows That Presented a CL at 21 and 40 days Postpartum According to Ultrasound Examination

**FIGURE 5:**
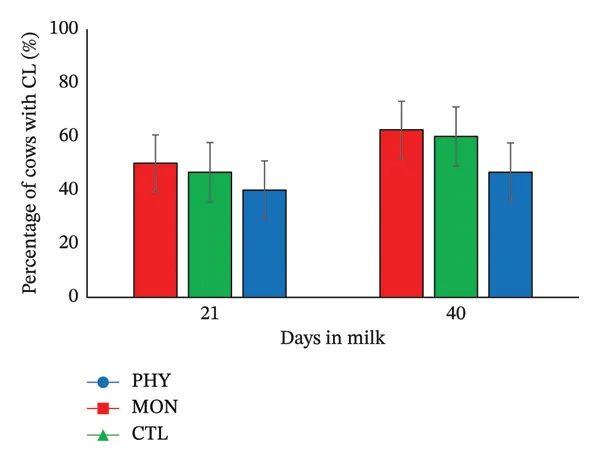
Percentage of cows that presented a corpus luteum (CL) at 21 and 40 days postpartum according to ultrasound examination in cows in the mix of phytochemical (PHY), monensin (MON), and control (CTL) groups.

Figure 5 shows the average percentage of cows (primiparous and multiparous) that presented a CL at Day 40 postpartum, being 62% in the MON, 54% in the CTL, and 46% in the PHY ±10% (mean ± SEM). At 21 days postpartum, the percentage of cows with a CL was 57%, 49%, and 46% ±13% for the MON, CTL, and PHY groups, respectively. At 40 days postpartum, these values increased to 68% for MON, 60% for CTL, and remained at 46% for PHY ±13% (mean ± SEM).

## 4. Discussion

As far as we know, this is the first study investigating the effect of phytochemical mix components in an integrated approach including milk production, metabolic and endocrine profiles, ovarian postpartum cyclicity, and hematology in primiparous and multiparous dairy cows.

The use of additives (PHY and MON) improved milk production in primiparous cows. Cows supplemented with PHY produced 8.3% more milk than the control group, while those supplemented with MON produced 7.4% more than controls. This effect was not observed in multiparous cows, where PHY‐supplemented animals tended to produce 4.7% less milk than those supplemented with MON, with no significant differences compared with the control group. Several recent studies, including the meta‐analysis conducted by Daning et al. [24], suggest that supplementation with essential oils at doses of 0.5–1.0 g/cow/day does not affect milk yield. However, Braun et al. [14] reported increased milk production using a blend of eugenol, menthol, and anethole at a dose of 1.2 g/cow/day. These findings are consistent with those of Kung [25], who observed increased milk production from early to mid‐lactation when supplementing a mixture of essential oils (CRINA) at 1 g/cow/day. The differences among studies are likely due to variations in the type of oils used compared with the present study. Several studies have shown that primiparous cows experience greater physiological and metabolic challenges during the transition period compared with multiparous cows, as milk production is subordinate to growth [21, 26]. This increased vulnerability may explain their differential response to additive supplementation, which could contribute to mitigating the metabolic demands of the transition period. In contrast, multiparous cows did not show productive improvements with supplementation, possibly due to their greater adaptive capacity at the onset of lactation. Notably, primiparous cows that received MON showed indicators of more efficient energy utilization during the transition. In a study parallel to ours, Roche et al. [27] demonstrated that this group was characterized by lower DMI in the prepartum period and better postpartum body condition, a pattern consistent with previous studies that associate MON supplementation with a better energy balance [28].

Energy balance indicators (BCS, NEFA, and BHB) showed that primiparous cows supplemented with MON had better body condition compared to control group. However, this difference was not reflected in lipid mobilization, as NEFA and BHB concentrations did not differ between treatments in any of the parity groups. The trajectory of body condition throughout the transition period and the onset of lactation remained within the expected range, with all cows starting at a score between 3.0 and 3.5 at the beginning of the study and experiencing a loss of approximately 0.5 points postpartum [27]. Similarly, NEFA and BHB concentrations remained within the physiological thresholds considered acceptable for the transition and early lactation periods (prepartum: NEFA < 0.4 mmol/L and BHB < 0.46 mmol/L; postpartum: NEFA < 0.6 mmol/L and BHB < 0.8 mmol/L; [28], suggesting that the animals did not experience critical states of energy imbalance and received an adequate nutrient supply to meet their requirements at the onset of lactation, which could explain why the additives did not exert greater effects. Previous studies have shown that certain additives, such as plant extracts or essential oil blends, can reduce serum concentrations of these metabolites [15, 16]. However, variability in the compounds used, the doses applied, treatment duration, physiological stage during administration, and the production system (confinement vs. mixed grazing) may explain the discrepancies observed across studies. Although all cows showed values below the established thresholds, primiparous cows exhibited higher NEFA and BHB concentrations than multiparous cows, suggesting a greater negative energy balance, possibly associated with their ongoing growth. This may be due to different nutrient partitioning compared with multiparous cows, prioritizing energy for growth and body reserves instead of allocating it entirely to milk synthesis. Additionally, under grazing conditions, primiparous cows may be more affected by forage access competition, as reported by Meikle et al. [29], and by a slower adaptation to grazing, with lower grazing activity and bite rates during pasture access [30].

Another indicator used to assess energy balance was cholesterol. In primiparous cows, a difference in cholesterol concentrations was observed from the second week postpartum, with higher levels in the MON group compared with the PHY and CTL groups. This finding is consistent with previous studies [31], in which MON, at the same dose used in the present study, resulted in higher cholesterol concentrations than in the control group. This is likely explained by the fact that cows in the MON group had a better energy balance, mobilizing less fat from adipose tissue (better body condition), which could lead to reduced hepatic fat accumulation, improved liver function, and consequently, greater production of triglycerides and cholesterol [32]. Another hypothesis could be that cows with MON have a greater intestinal absorption of cholesterol [33–35]. In all treatments, cholesterol concentrations decreased before calving and began to increase afterward. This increase observed in all animals is consistent with the literature and is associated with higher DMI [36, 37]. The decrease in glucose during the peripartum period is consistent with previous studies, as metabolism prioritizes its use in the mammary gland for lactose synthesis [29]. This decrease in glucose concentrations, due to increased demand and the lower DMI, also leads to a decrease in insulin levels, as an homeorhetic adaptation to increase the availability of energy substrates in response to NEB during this period, resulting in more glucose being allocated to the mammary gland [21]. Cinnamaldehyde is known to stimulate insulin synthesis by the pancreas, thereby increasing insulin sensitivity in peripheral tissues. In this context, it could be expected that cows supplemented with PHY (which contains this essential oil) would have higher insulin concentrations and improved glucose utilization by peripheral tissues, thereby reducing negative energy balance without necessarily changing circulating glucose levels [38]. However, in the present study, no differences were observed between treatments. This could be explained by the fact that the study by El‐Azrak et al. [38] was conducted in goats and that cinnamaldehyde was administered together with menthol and thymol, which act synergistically.

Calcium, phosphorus, and the calcium/phosphorus ratio also did not differ between treatments in both primiparous and multiparous cows. This is consistent with Mezzetti et al. [39], who found no difference in calcium with MON administration. Regarding mineral metabolism, the reduction in calcium during the peripartum period is a normal physiological process attributed to greater uptake by the mammary gland for milk production [40]. Additionally, multiparous cows had lower calcium concentrations than primiparous cows, which can be explained by the higher demand for this mineral to sustain high milk production.

Regarding the immune response, in multiparous cows, the MON group showed greater monocyte counts compared with the control group. This increase exceeded reference values and occurred between week −1 and week 1 postpartum. During the transition period, monocytes exhibit a greater capacity to respond to inflammatory stimuli, resulting in increased cytokine production [41]. We would have expected the phytochemicals to result in lower leukocyte counts, as they exert modulatory effects on monocytes and macrophages, helping to limit excessive monocytosis induced by bacterial infections. Supporting this hypothesis, Escalera‐Moreno et al. [42] reported that, in newly weaned calves, the use of polyphenols (the extract used in PHY) reduced the concentrations of inflammatory markers such as IL‐6 and haptoglobin and decreased the surface expression of CD14 on monocytes, suggesting that these compounds may help attenuate the negative effects of sustained immune system activation. Complementarily, de Mello et al. [13] observed lower blood leukocyte counts in cows fed phytobiotics, associated with a reduction in lymphocytes, indicating that these additives may modulate inflammatory processes and exert an anti‐inflammatory effect. Although a decrease in leukocyte/lymphocyte counts could also be interpreted as immunosuppression, the productive outcomes and blood profiles reported in the present study support the hypothesis that this reduction primarily reflects an immune‐regulatory effect. Reinforcing this interpretation, Leal et al. [43] reported that the anti‐inflammatory actions of phytochemicals are linked to reduced pro‐inflammatory cytokines and increased release of IL‐10, which plays a key role in containing excessive immune responses. Although leukocyte counts in cows supplemented with MON did not show significant differences compared with the other treatments, total leukocyte, monocyte, lymphocyte, and neutrophil values were above the reference ranges, which could suggest a greater degree of immune system activation during the transition period.

Regarding the red blood parameters, all parameters remained within reference ranges [23]. However, cows supplemented with MON showed higher percentages of HCT, MCV, and MCH. These results are consistent with those reported by Drong et al. [44], who observed that animals with better BCS had higher HCT percentages. Similarly, Uyarlar et al. [16] reported no effect on erythrocyte parameters when administering an oil mixture (Digestarom™ Dairy®) at a dose of 3 g/day per animal [45]. On the other hand, in primiparous cows supplemented with additives, who also had higher milk production, lower concentrations of total RBC and HGB were observed, like the findings of Kumar and Pachauri [46], who reported that total RBC and HGB tend to decrease as milk production increases.

Regarding the resumption of ovarian cyclicity, no significant treatment effect was observed on any of the variables analyzed. Overall, 60% of the cows exhibited cyclicity 40 days postpartum. This finding does not agree with our initial hypothesis, since we expected that the additives (PHY and MON), by modifying rumen fermentation and increasing propionate production and consequently the availability of glucose and energy, would promote an earlier resumption of ovarian activity. Our results are consistent with those reported by Duffield et al. [44] who also found no effect of MON on reproductive parameters. No bibliographic references were found regarding the use of phytochemicals and the resumption of postpartum ovarian cyclicity.

## 5. Conclusion

In conclusion, on grazing conditions, supplementation with PHY and MON improved milk production in primiparous cows, while no benefits were observed in multiparous cows. The additives did not affect endocrine or metabolic parameters, except that MON increased serum cholesterol concentration in primiparous cows. Treatments did not significantly affect hematological parameters or the resumption of ovarian activity. All of this suggests that proper herd management (especially feeding) may prevent the additives from having greater effects.

## Funding

This research was partially funded by CSIC (Sectoral Commission for Scientific Research, Universidad de la República) and CCPA Group (Janzé, France).

## Conflicts of Interest

The coauthors (Aline C. Dall‐Orsoletta and Marisela Arturo‐Schaan) are employees of CCPA group, which partially financed the project and provided the phytochemical product tested. The other authors declare no conflicts of interest.

## Data Availability

The datasets used and/or analyzed during the current study are available from the corresponding author upon request.
